# Supplementary material for: In vivo fate of free and encapsulated iron oxide nanoparticles after injection of labelled stem cells
Source: Nanoscale Adv. 2018 Sep 25;1(1):367–77. doi: 10.1039/c8na00098k (PMC9473218; doi:10.1039/c8na00098k)
Supplement: NA-001-C8NA00098K-s001 [file NA-001-C8NA00098K-s001.pdf]

Electronic Supplementary Material (ESI) for Nanoscale Advances.
This journal is © The Royal Society of Chemistry 2018

In vivo fate of free and encapsulated iron oxide nanoparticles after injection of labelled stem cells

Sumaira Ashraf[1*], Arthur Taylor[2,3], Jack Sharkey[2,3], Michael Barrow[4], Patricia Murray[2,3], Bettina Wilm[2,3], Harish Poptani[2,3], Matthew J. Rosseinsky[4], Dave J. Adams[4,5], Raphaël Lévy[1*]

[1]Department of Biochemistry, Institute of Integrative Biology (IIB), University of Liverpool, Liverpool, United Kingdom

[2]Department of Cellular and Molecular Physiology, Institute of Translational medicine (ITM), University of Liverpool, Liverpool, United Kingdom

[3]Centre for Preclinical Imaging, Institute of Translational medicine (ITM), University of Liverpool, Liverpool, United Kingdom

[4]Department of Chemistry, University of Liverpool, Liverpool, United Kingdom

[5]School of Chemistry, University of Glasgow, Glasgow, United Kingdom

[*]**Corresponding authors:** rapha@liverpool.ac.uk, sumaira@liverpool.ac.uk

Supplementary Information

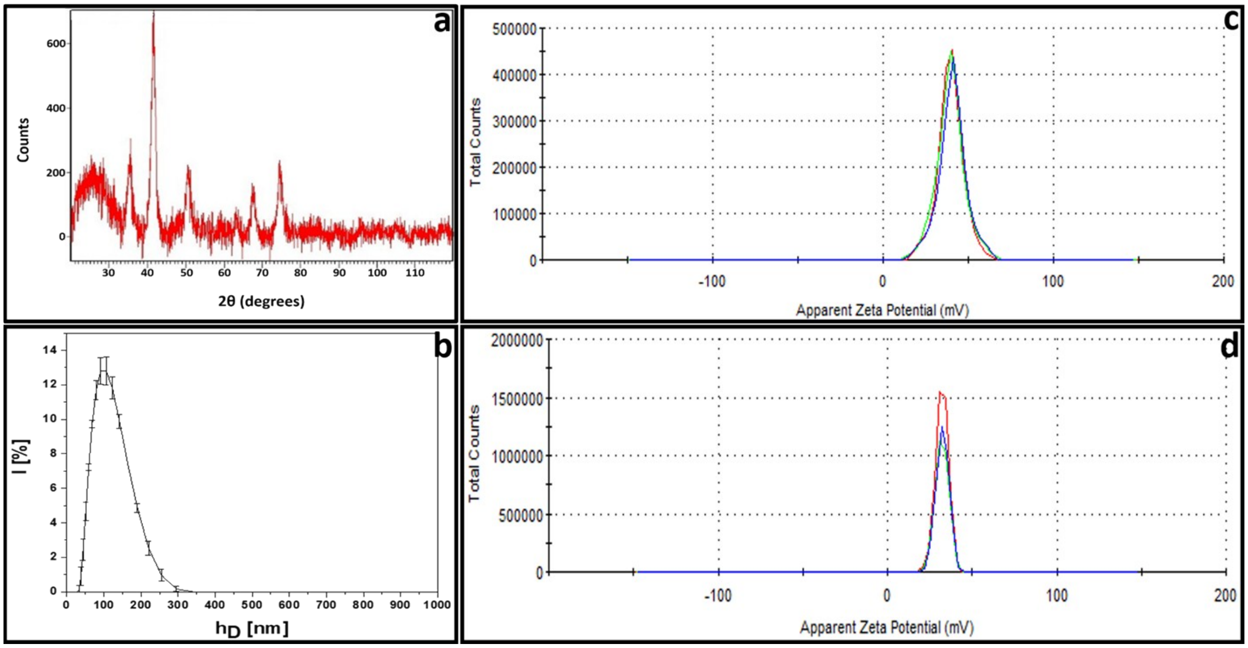

Figure SI.1: *Characterization of free and encapsulated SPIONs by means of powder X-ray diffraction (pXRD), dynamic light scattering and zeta potential (ZP). pXRD of free SPIONS (a), hydrodynamic diameter (% intensity) of free SPIONs in water (b), and zeta potential measurements of free (39.5 mV) and encapsulated SPIONs (32.1 mV) in water is presented in (c) and (d), respectively. Three repeat measurements are shown for (c) and (d). The ZP measurements for large sedimenting objects (encapsulated SPIONs; d) is not reliable as sedimentation during the measurement period affects the accuracy of the data.*

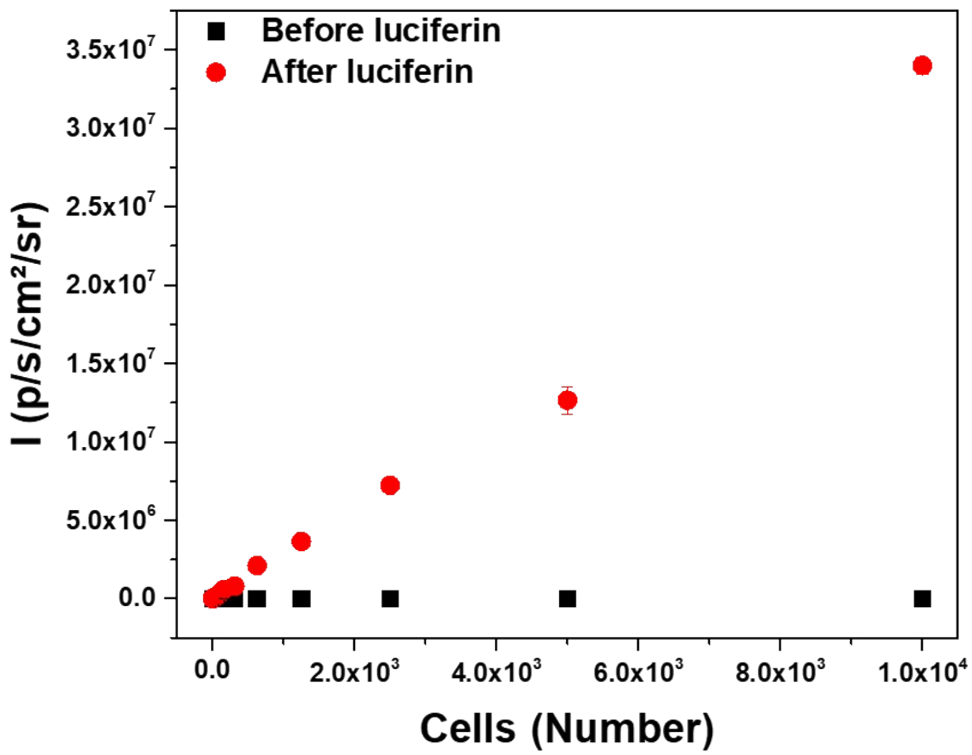

Figure SI.2: *In vitro bioluminescence of mMSCs before and after the addition of D-luciferin.*

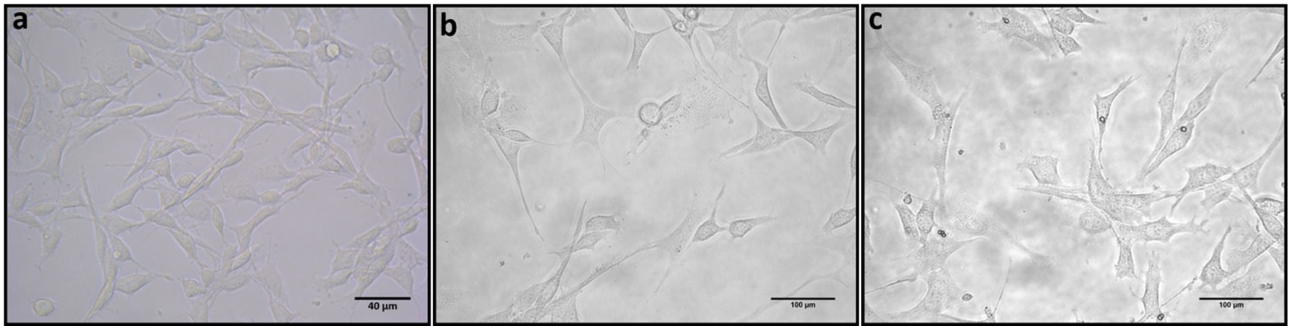

Figure SI.3: *Morphology of mMSCs after labelling with particles. Unlabelled cells (a), cells labelled with free (b) and encapsulated SPIONs (c). After trypsinization the particle labelled cells were kept in ice cold PBS for 5 - 6 hrs and re-seeded. Cells were observed 3 days post seeding using 40 and 20 x objectives for unlabelled and particle-labelled cells, respectively.*

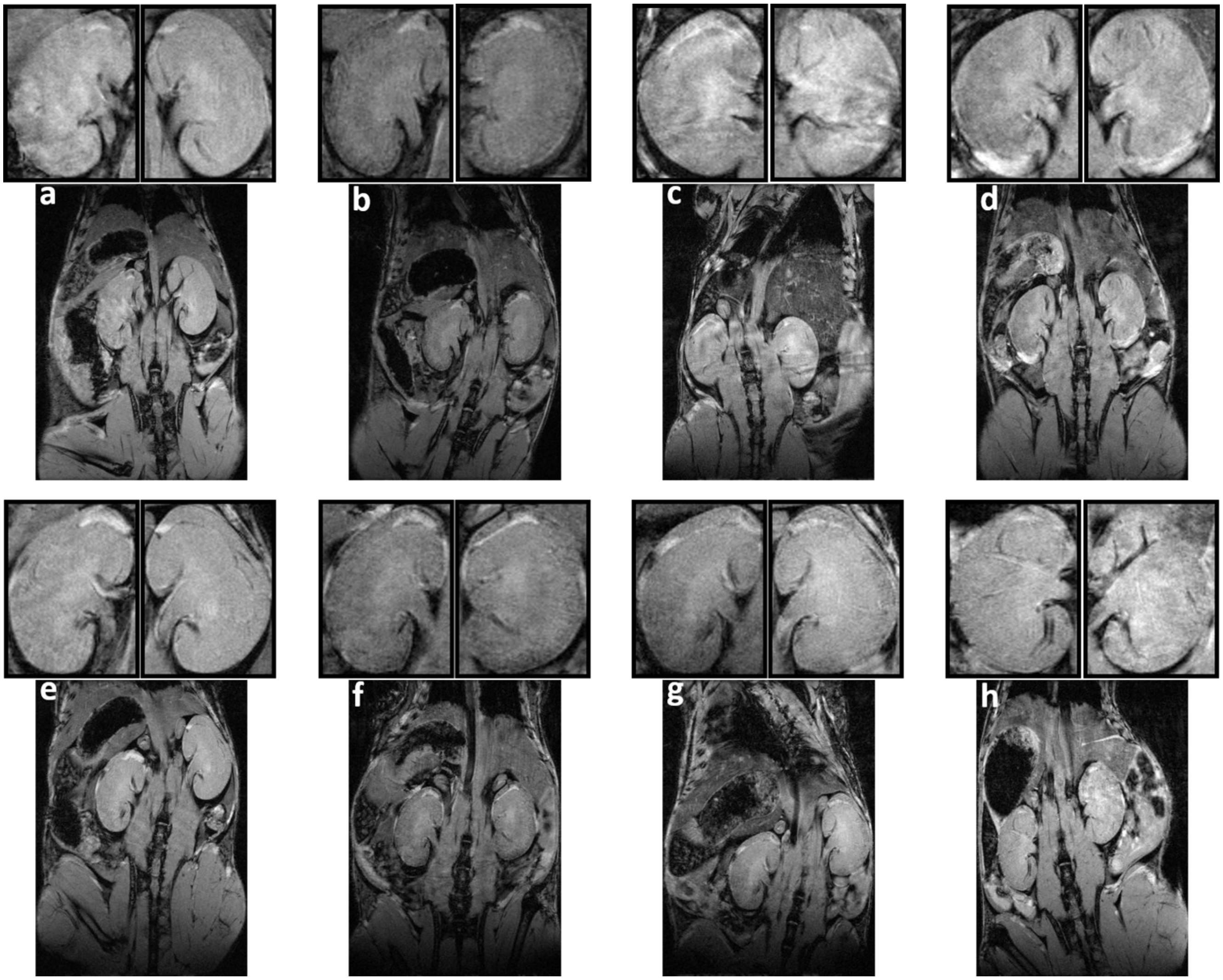

Figure SI.4: *MR images of mice organs at selective days are presented. For clarity one mouse representing each treatment group (free SPIONs; a-d, and encapsulated SPONs; e-h) are presented with zoomed in kidneys above each mouse (to see the contrast of SPIONs in kidney cortices) are shown. Mice abdominal MR scans before injection (baseline; a, e), day 0 (injection day; b, f), day 1 (post injection; c, g) and day 14 (post injection; d, h) are presented. Detailed abdominal scans of all mice at each imaging/scanning day can be found from* https://doi.org/10.5281/zenodo.1203991.

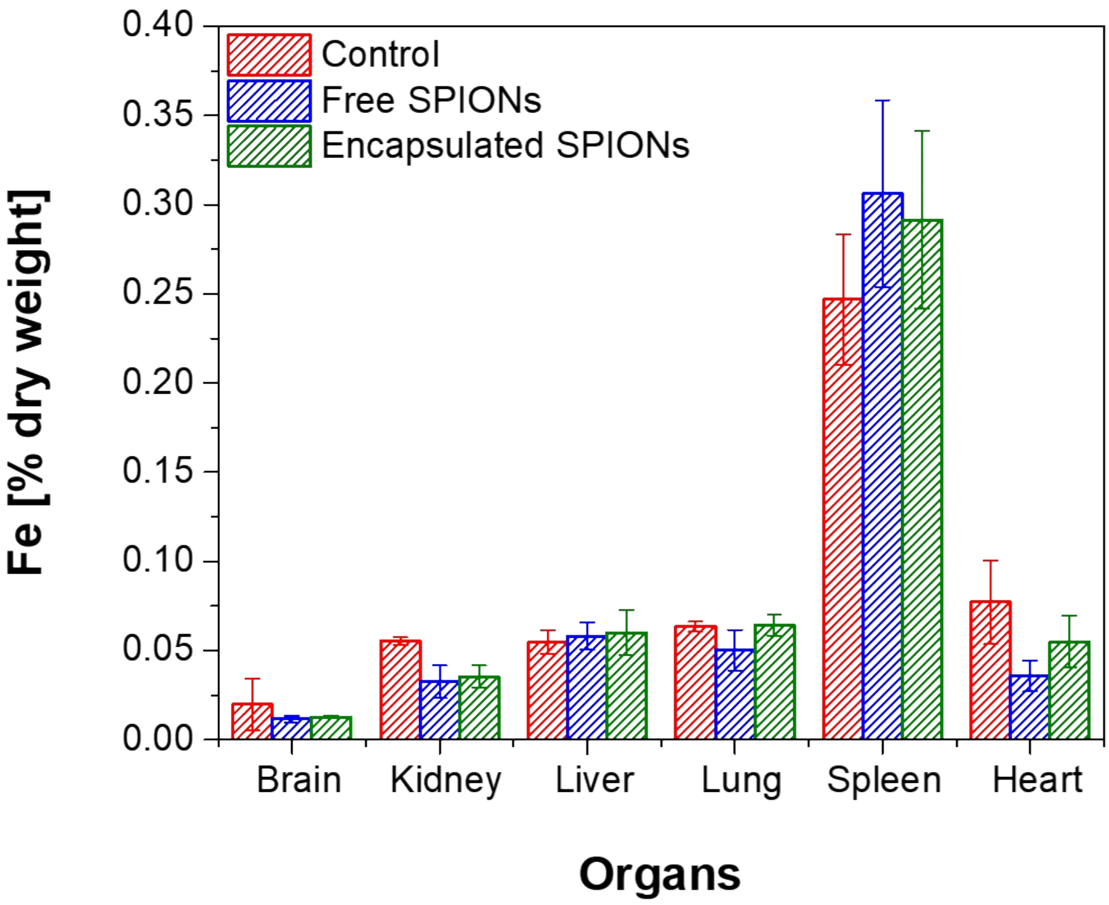

Figure SI.5: *ICP-OES based Fe (iron) quantification of dried organs, collected at the end of experiments (day 14 post injection). Control group of mice did not receive any injection.*

**Sequence**	**FLASH T2***	**B0 Map**	**MGE**<br>**T2* Map**
Purpose	T2* imaging	T2* Map<br>Shimming	T2* relaxation
Echo time (ms)	4.9	1.59	4.5
Repetition time (ms)	253	10	800
Flip angle	20	15	50
Matrix size (pixels)	326 x 326	64 x 64	256 x 256
Image resolution	92 µm/pixel	625 µm/pixel	117 µm/pixel
Field of view (mm)	30 × 30	40 × 40	30 × 30
Number of averages	8	3	2
Slices	10	1	1
Slice thickness (µm)	500	40000	1000
Echo images	N.A	2	8
Echo spacing (ms)	N.A	3.569	5.5

Acquisition time	3 min, 13 sec, 915 ms	2 min, 2 sec, 880 ms	8 min, 32 sec

Table SI.1. MR acquisition parameters for SPIONs based phantoms. All images and mapping were axially performed.

**Sequence**	**T2 Map MSME**	**B0 Map**	**MGE T2* Map**	**RARE T2**	**FLASH (axial and coronal)**
Purpose	T2 relaxation	Shimming	T2* relaxation	T2 imaging	T2* imaging
Echo time (ms)	9	1.59	4.5	36	7
Repetition time (ms)	2000	10	800	2200	330
Flip angle	N.A	15	50	N.A	30
Matrix size (pixels)	256 x 256	64 x 64	256 x 256	512 x 512	512 x 512
Image resolution	117 µm/pixel	625 µm/pixel	117 µm/pixel	59 µm/pixel	59 µm/pixel
Field of view (mm)	30 × 30	40 × 40	30 × 30	30 × 30	30 × 30
Number of averages	1	3	2	6	6
Slices	5	1	1	19	19
Slice thickness (µm)	1000	40000	1000	500	500
Echo images	25	2	8	1	N.A
Echo spacing (ms)	9	3.569	5.5	12	N.A

Acquisition time	8 min, 32 sec	2 min, 2 sec, 880 ms	5 min, 7 sec, 200 ms	14 min, 4 sec, 800 ms	16 min, 53 sec, 760 ms
Rare factor	N.A	N.A	N.A	8	N.A
Excitation angle	90	N.A	N.A	90	N.A
Refocusing angle	180	N.A	N.A	180	N.A

Table SI.2. MR acquisition parameters for labelled mMSCs based phantoms.

**Sequence**	**FLASH T2***	**B0 Map**	**MGE T2* Map**
Purpose	T2* imaging	Shimming	T2* relaxation
Echo time (ms)	5.5	1.59	4.5
Repetition time (ms)	305	10	1050
Flip angle	20	15	50
Matrix size (pixels)	386 x 386	64 x 64	256 x 256
Image resolution	91 µm/pixel	703 µm/pixel	137 µm/pixel
Field of view (mm)	35 × 35	45 × 45	35 × 35
Number of averages	3	3	2
Slices	23	1	23
Slice thickness (µm)	500	45000	500
Echo images	N.A	2	8
Echo spacing (ms)	N.A	2.855	4.5
Acquisition time	5 min, 53 sec, 190 ms	2 min, 2 sec, 880 ms	6 min, 43 sec, 200 ms

Reconstruction MAP<br><br>Signal to noise	N.A	20	N.A

Table SI.3. MR acquisition parameters for *in vivo* MRI.
